# Improving health-related quality of life instrument translation into South African languages

**DOI:** 10.4102/safp.v63i1.5361

**Published:** 2021-11-08

**Authors:** Sophia E. Marsh, Ilse Truter

**Affiliations:** 1 Department of Pharmacy, Faculty of Health Sciences, Nelson Mandela University, Gqeberha, South Africa

**Keywords:** cost-utility analysis, economic evaluation, health-related quality of life, health technology assessment, South Africa, translation

## Abstract

**Background:**

Most health-related quality of life (HRQoL) instruments have been created in English, which can influence their reliability and validity in non-English speaking populations. This study assessed the translation methodology of HRQoL instruments that have been used and translated into South African languages and which could be applied in cost-utility analyses (CUAs).

**Methods:**

A 2019 systematic review was updated with searches conducted in Medline, the Web of Science^TM^ (WoS^TM^) Core Collection and the South African SciELO collection via the WoS^TM^ Platform. Additional searches in Sabinet’s African Journals database and on instrument developers’ webpages were performed. Only HRQoL instruments suitable for CUAs were included. Articles reporting at least one element of the translation methods were included. Established good practice principles were used to evaluate the translation methodology.

**Results:**

Within the 39 publications identified, a dozen translated instruments have been used in South Africa. All instruments used were translated from English and none had originally been created in South Africa. Instrument developers’ translations were used more than study investigators’ translations. Almost all instrument developer versions met the full translation criteria. No investigator translated instrument met the full translation criteria primarily because recommendations on forward and back translations were not followed. However, this analysis was hampered by a lack of methodological reporting details. The most used instruments, which also had the most translated versions available, were the EQ-5D-3L, SF-36 version 2 and EORTC QLQ-C30.

**Conclusion:**

Instrument developers’ translations more often met recommended translation methodology compared with investigators’ versions. The EQ-5D-3L may be best suited for South African economic evaluations and for use in clinical practice, but further work may be needed.

## Introduction

Patient reported outcomes measures (PROMs) can be applied in diverse settings to guide the choice of healthcare interventions. It does so not only by providing data on the clinical outcomes with a health technology or the disease course, but because it can also be combined with cost data in economic evaluations to determine the cost-effectiveness thereof. Measuring provider performance and quality of services using PROMs, the use of health technology assessment (HTA) and economic evaluations will play an increasingly important role in South African private healthcare provision and national public health policy decisions.^1,2,3,4^ Specifically, under National Health Insurance (NHI), economic evaluations as part of a more formal HTA programme could be used to determine the cost-effectiveness of treatments provided as part of the NHI fund. On this point the 2019 NHI Bill stated that:

The Ministerial Advisory Committee on Health Technology Assessment for National Health Insurance, which must be established to advise the Minister on Health Technology Assessment … must regularly review the range of health interventions and technology by using the best available evidence on cost-effectiveness, allocative, productive and technical efficiency and Health Technology Assessment.^1^ (p. 29)

Such cost-effective evaluations frequently use health-related quality of life (HRQoL) data in cost-utility analyses (CUAs) because it allows for comparisons across health situations and programmes. These analyses can subsequently be employed for making resource allocation decisions. Indeed, this is the intention of the 2021 draft HTA guideline issued for comment by the National Department of Health as part of the Essential Drugs Programme’s appraisal of medicine informing their inclusion on to the National Essential Medicines List (NEML)^5^: the guideline proposes that a CUA be conducted if a comprehensive cost-effectiveness evaluation is required.

However, not all HRQoL data are suitable for CUAs. Data from HRQoL instruments should be generated by a PROM that is a generic instrument because it assesses the treatment outcomes across a range of populations and interventions. Such instruments should also measure attributes across multiple health domains, have a health state classification system based on the combination of responses and the health states should have associated numeric values that represent the patient or general public preference for each health state generated by the instrument.^6^ For this reason, they are also called multi-attribute utility instruments (MAUIs) or preference-based measures. It is possible to convert the results from some non-MAUIs/preference-based measures into generic HRQoL data that can be used in CUAs, but this should be avoided if possible because of the limitations thereof.^6,7^

Whether for clinical decisions, economic evaluation or health policy decisions, it is important that PROMs are reliable and valid in the target population. But this can be influenced by instrument language (amongst others): if English language measurements are used in non-English speaking populations, their understanding of the questions and responses given may not correspond to the intended concepts.^8^ The importance of adequate translations into South African languages is emphasised by the findings of studies that evaluated the validity, reliability and cultural adaptation of translated HRQoL instruments. Researchers found that reliability and validity were influenced by socioeconomic factors such as education, literacy and rural or urban living, which were often associated with populations’ cultural background and historical racial inequalities.^9,10,11,12^ In addition, understanding could be impacted when no equivalent word existed in the South African language,^13^ or because of difficulty in transferring English concepts into African culture.^11,14^ Consequently, the issues identified required the researchers to make semantic and conceptual changes to the instruments. Changes in delivery of some instruments such as an oral explanation of the nature of the instrument and questions or requesting permission to ask questions considered sensitive in a particular cultural group were also necessary to alleviate participants’ discomfort in completing the questionnaires. Therefore, by using an inadequate translation, the interpretation of the results and conclusions generated by it may be limited. Moreover, inadequately translated instruments impact a study’s generalisability to the wider population. This in turn may cause uncertainty when such results are needed to inform the priority setting process, thereby potentially delaying patients’ access to new technologies.^15^ Therefore, to optimise patient outcomes and support patients’ access to technologies, HRQoL instruments should be administered in the target population’s home language(s). If such a language version is not available, they should be translated according to good translation principles as this will give greater certainty in the results obtained. Unsurprisingly, the South African Guidelines for Pharmacoeconomic Submissions (SAGPS), point out that all HRQoL instruments should be validated using South African data and request detailed information supporting the validity of the tool in the South African context.^16^ Similarly, the draft HTA guideline for the NEML proposes that HRQoL for a CUA should be measured from a representative sample of the South African population using a validated instrument, and the effects should be valued with a South African-based value set^5^ (a value set is a single score based on the South African general population’s preference for different health states possible with a MAUI). However, whilst a systematic review found that a body of HRQoL data exists in South Africa, details on the instrument language versions used or the translation methods employed were reported infrequently.^17^

This study therefore sought to (1) provide a list of HRQoL instruments suitable for CUAs that have been used in South Africa using one or more South African languages (other than English), (2) critique the methods used to produce the translated versions, and (3) make recommendations on which instruments may be suitable for future HRQoL measurement within the context of conducting CUAs in South Africa as part of national HTA.

## Methods

### Study design

The study forms part of a broader project that aimed to identify evidence and research gaps in HRQoL data in South Africa against the background of national HTA and CUA; and for which a systematic review was conducted in January 2019. The methods and results of this bigger project have previously been published.^17,18^ The current analyses build on the earlier systematic review through updated and new searches and new qualitative and quantitative analyses focussing on the translation methods used in the identified studies.

### Search strategy and inclusion criteria

Briefly, the 2019 systematic review included full text articles and abstracts that reported South African- based HRQoL research using HRQoL instruments suitable for CUAs, namely MAUIs and instruments that can be mapped to it. There were no publication time restrictions and studies of any design were included. Multiple literature databases were searched, combining keywords and free text in the title and abstract and subject heading terms. The 2019 review was revisited to identify multi-country studies that were originally excluded because the publications reported the use of a MAUI, but did not report South African results separately. This allowed the inclusion of all publications that reported the use of translated instruments in the current analysis, regardless of whether the South African cohort results were reported.

### Data sources

Searches covering 2019 onwards were conducted in the Web of Science^TM^ (WoS^TM^) platform on 11 April 2021 as per the 2019 review: the databases searched were Medline, the WoS^TM^ Core Collection and the South African SciELO collection. As reference list searching in the original review identified a small number of articles from South African specific journals not indexed on any of the databases included in the WoS^TM^ platform, additional keyword and free-text searches were conducted on Sabinet’s African Journals database to account for non-indexed South African journals.

### Screening, inclusion criteria and information extraction

In the 2019 systematic review, two reviewers were responsible for first and second pass screening and data extraction into Excel^®^, whereas only one reviewer (the first author) screened articles and extracted the data from the updated searches. In addition to the criteria used in the 2019 review, articles were excluded if they did not contain information that identified the instrument as a South African language version. Instruments created in South African languages were included, but studies and their associated publications were excluded if only English language instruments were used. Furthermore, translated instruments available from, or endorsed by, the original instrument developer were considered for inclusion as were instruments translated by investigators for their specific study without input from the original instrument developer. However, only publications reporting that the instrument developer’s translated and endorsed version was used and publications by investigators that reported at least one of their translation stages, were selected (see Table 1). Finally, multiple publications of the same study were marked as ‘duplicate’ and only the one reporting the most detailed translation methodology were retained. For instrument developer versions, their webpages and the Mapi Research Trust’s PROQOLID^TM^ database were searched for information on the translation methods. In some instances, written requests were sent to the instrument developers or their appointed representatives to confirm availability of translated South African language instruments and to request information on translation methods.

**TABLE 1:** Recommended criteria for translation methods used in the assessment.

Stage	Description
Forward translation	Professional translators, who are native speakers of the target language and fluent in the instrument source language, independently conduct at least two parallel translations of the original instrument into the target language. This enables detection of errors and divergence of conceptual meaning.
Reconciliation and consensus	Translations are synthesised by comparing and merging the forward translations into a single translation, creating a consensus version. Approaches may differ, but ideally this should be performed by a committee, an independent native speaker of the target language not previously involved in forward translation or in-country investigator who may have prepared one of the forward translations.
Back translation	A quality control step whereby professional translators who are native speakers of the source language and fluent in the target language conduct at least two independent translations of the reconciled consensus version back into the instrument’s source language. This ensures that the same meanings have been derived in the translated version and avoids having a different conceptual basis to the source measure.
Review and harmonisation	Another quality control step whereby the back translated versions are reviewed by a committee, or the project manager and the back translators, or the project manager and key in-country consultants, against the original document. This aims to detect and deal with translation discrepancies between the different language versions and supports production of a conceptually equivalent version. Thereafter, the pre-final version is produced.
Pilot and cognitive debriefing	Lay people or a sample of the target population test the comprehensibility and equivalence of the pre-final version through soliciting feedback on the understandability, interpretation and cultural relevance of the translated instrument.
Finalisation	Results from the cognitive debriefing are incorporated into the translation, which is proofread and finalised by a committee or the project manager and a key in-country person.

*Source:* Guillemin F, Bombardier C, Beaton D. Cross-cultural adaptation of health-related quality of life measures: Literature review and proposed guidelines. J Clin Epidemiol. 1993;46(12):1417–1432. https://doi.org/10.1016/0895-4356(93)90142-N; Wild D, Grove A, Martin M, et al. Principles of good practice for the translation and cultural adaptation process for Patient-Reported Outcomes (PRO) measures: Report of the ISPOR task force for translation and cultural adaptation. Value Health. 2005;8(2):94–104. https://doi.org/10.1111/j.1524-4733.2005.04054.x

### Assessment criteria

The final set of publications were evaluated against the guidelines for cross-cultural adaptation of HRQoL measurements first outlined by Guillemin et al.,^8^ and the International Society for Pharmacoeconomics and Outcomes Research (ISPOR) Principles of Good Practice for the translation and cultural adaptation process for PROMs.^19^ These best practice recommendations were selected because Guillemin et al. has been used most often in the reported literature, and the ISPOR publication by Wild et al. is frequently referenced as a recognised methodology within the pharmaceutical industry and by the United States Food and Drug Administration. The classification of each stage as outlined in Table 1 was scored as: positive (+): procedure performed according to the quality criteria used; negative (–): procedure not performed as recommended; uncertain (?): insufficient information available to rate the stage; unknown (0): no information available to rate the stage.

### Ethical considerations

The Faculty Postgraduate Studies Committee at the Nelson Mandela University reviewed the study proposal and granted ethics approval (ethics clearance reference number: H18-HEA-PHA-009).

## Results

### Literature retrieved

**FIGURE 1:**
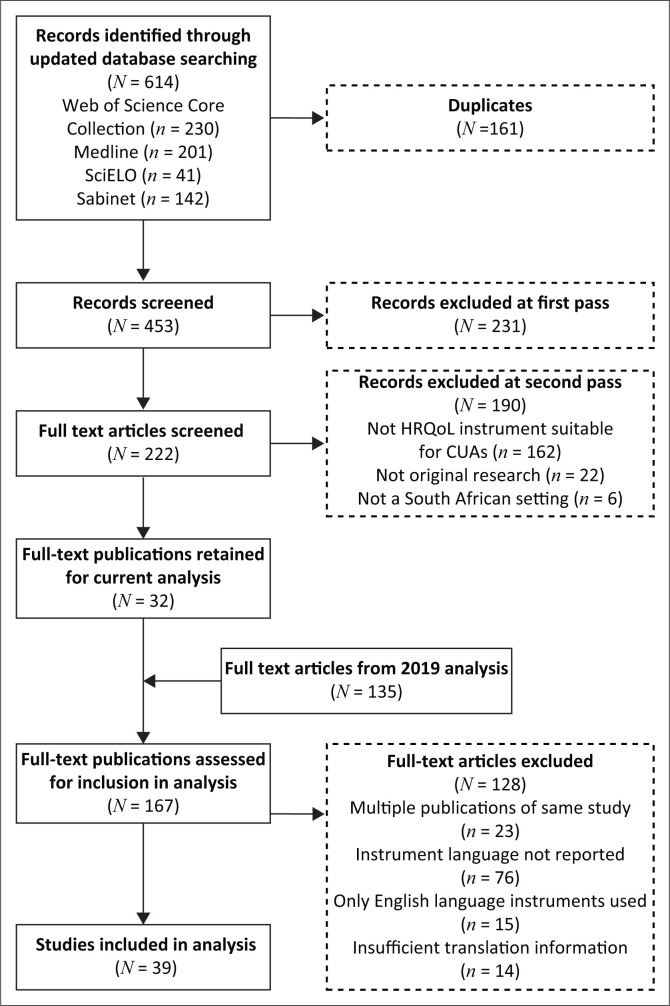
Publication identification, retrieval and inclusion flow diagram.

The updated literature searches identified an additional 614 publications for screening, of which 32 were retained. The 2019 review consisted of 123 articles and together with a further 12 articles, which were originally excluded as no study results were reported, constituted the bulk of the articles. Thus, 167 publications were assessed for their instrument translation methodology. Of these, 128 were excluded because they did not report the instrument language (*n* = 76), reported on the same study (*n* = 23), used only English language instruments (*n* = 15), and contained no description of the translation methods or information on whether the translated version was obtained from the instrument developer (*n* = 14). Thus, the remaining 39 studies were critiqued. The flow of articles identified and included in the analysis is illustrated in Figure 1.

### Instruments identified

Three new instruments were found that had not been identified in the 2019 review: the Asthma Quality of Life Questionnaire, Women’s Health Questionnaire and Parkinson’s Disease Questionnaire. However, only the Parkinson’s Disease Questionnaire provided information on the language version used. This instrument, and the other included instruments are listed in Table 2. The table describes the translated HRQoL instruments suitable for CUA that have been used in South Africa, the language versions used in the studies and the language versions currently available from the instrument developer. It shows that in some instances where there was an absence of instrument developer translated versions, the investigators created their own versions. In addition, currently most instruments listed have at least one South African language version available from the developer (other than English). However, none are available in all South African languages but the EQ-5D-3L has the most translated versions available from the developer. In more than a third of the studies using generic instruments (which are suitable for a range of diseases), people living with the human immunodeficiency virus (HIV) formed the target population. The remainder of the studies using generic instruments included people with gastroenterological conditions, musculoskeletal conditions, unspecified or multiple chronic conditions and tuberculosis.

**TABLE 2:** Translated health-related quality of life instruments that have been used in South Africa.

Instrument and version	Instrument description	Language version(s) used in the published studies included in the review	South African language version(s) currently available from the instrument developer
**Generic instruments**			
AQOL-6D	Originally designed to measure HRQoL in economic evaluation studies, it consists of six domains that measure independent living, mental health, coping, relationships, pain and senses.	Afrikaans	None
		Setswana	
**EQ-5D**			
3L	An instrument for use in clinical and economic appraisal and population health surveys, it assesses health status across five dimensions with three response levels: mobility, self-care, usual activities, pain/discomfort and anxiety/depression.	Afrikaans	Afrikaans
		isiXhosa	isiXhosa
		isiZulu	isiZulu
		Setswana	Northern Sotho†
		Sesotho	Sesotho
			Setswana
			Tsonga
5L	The 5-level version of the instrument was introduced to improve the instrument’s sensitivity and to reduce ceiling effects.	isiXhosa	Afrikaans
			isiXhosa
			isiZulu
			Northern Sotho†
			Sesotho
			Setswana
HUI3	The instrument measures eight attributes: vision, hearing, speech, ambulation, pain, dexterity, smotion and cognition.	Afrikaans	Afrikaans
		Setswana	
PedsQL 4.0‡ Generic Core Scale (Standard, self-reported child and adolescent)	The 23-item instrument assesses HRQoL in children, adolescents and young adults with acute or chronic diseases. It can be self-reported or by a parent/proxy. It measures physical, emotional, social and school functioning.	Afrikaans	Afrikaans
		isiXhosa	isiXhosa
		isiZulu	isiZulu
		Sesotho	Sesotho
			Setswana
Satisfaction with Life Scale‡	A 5-item instrument designed to measure global cognitive judgements of satisfaction with life. It has been used in assessing subjective quality of life in people with various health conditions.	Afrikaans	Setswana§
		Setswana	
**SF-36 ‡**			
Version 1	The 36-item measures health status using eight scales, yielding two summary measures: physical and mental health. The physical measure includes physical functioning, role-physical, body pain and general health, whilst the mental health measures consist of vitality, social functioning, role-emotional and mental health. Version 1 became available in 1996 and version 2 in 1998, with version 2 offering increased range and precision, improved item wording and easy-to-use formats.	Afrikaans	None
		isiZulu	
Version 2		Afrikaans	Afrikaans
		isiXhosa	isiXhosa
		isiZulu	isiZulu
		Sesotho	Sesotho
			Setswana
SF-12‡	A reduced version of the SF-36, it produces similar results for physical and mental health scores with less respondent burden as it only has 12 items.	isiXhosa	Afrikaans
			isiXhosa
			isiZulu
			Sesotho
			Setswana
WHOQOL-BREF‡	A shorter version of the original WHOQOL-100 instrument, it aims to assess individuals’ perceptions on the quality of their life. It comprises 26 items, measuring four broad domains: physical health, psychological health, social relationships and environment.	Afrikaans	Afrikaans, however, although available from the WHO, it was not created by them
**Disease-specific instruments**			
DLQI‡	The instrument consists of 10 questions concerning patients’ perception of the impact of their skin diseases on different aspects of their HRQoL. It can be used in several skin diseases.	Afrikaans	Afrikaans
		isiXhosa	isiXhosa
		isiZulu	isiZulu
			Sesotho
			Setswana
EORTC QLQ-C30‡	A generic cancer questionnaire that covers the most common problems and symptoms of people living with cancer and measures overall health status and HRQoL.	Afrikaans	Afrikaans
		isiXhosa	isiXhosa
		isiZulu	isiZulu
		Setswana	Sepedi
			Sotho
			Setswana
**FACT ‡**			
General	A 27-item questionnaire designed to measure four domains in cancer patients: physical, social, emotional and functional well-being.	isiZulu	Afrikaans
		Sepedi	isiXhosa
		Setswana	isiZulu
			Sepedi
			Sesotho
			Setswana
Breast	The instrument consists of the FACT-General plus a breast cancer subscale, becoming a 37-item questionnaire focussing on five domains of HRQoL in breast cancer patients.	isiZulu	Afrikaans
		Sepedi	isiXhosa
		Setswana	isiZulu
			Sepedi
PDQ-39‡	The 39-item questionnaire assesses how often people affected by Parkinson’s experience difficulties across eight dimensions of daily living: mobility, activities of daily living, emotional well-being, stigma, social support, cognitions, communication and bodily discomfort.	Afrikaans	Afrikaans
		isiZulu	
		Setswana	

3L, 3-levels; 5L, 5-levels; AQoL-6D, Assessment of Quality of Life-6 domains; DLQI, Dermatology Life Quality Index; EORTC QLQ-C30, European Organisation for Research and Treatment of Cancer Quality of Life Questionnaire Core Questionnaire; FACT, Functional Assessment of Cancer Therapy; HUI3, Health Utility Index mark 3; PedsQL, Paediatric Quality of Life Inventory; PDQ-39, Parkinson’s Disease Questionnaire; SF, Short Form; WHOQOL-BREF, World Health Organization abbreviated version.

† , Listed as Northern Sotho by the developer, however, it is sometimes referred to as Sepedi, which is an official language whereas Northern Sotho is not named as an official language;

‡ , Requires mapping to a MAUI such as the EQ-5D;

§ , The investigator version became the instrument developer version.

### Translation of instruments

None of the measures were originally created in South African languages; all were originally English language instruments. Nearly all studies reported the use of HRQoL instruments to measure health outcomes, only six publications were methodological articles providing the results of testing an instrument’s reliability and validity or issues with its cultural adaptation.

Most studies (25/39) used the instrument developer’s translated version or created a version based on the developer’s translation manual and 14 studies used versions translated by the investigators. In three instances the researchers translated the instrument despite the availability of a translated version from the developer. The EQ-5D-3L was the most translated instrument (*n* = 15), followed by the SF-36 version 2 (*n* = 8) and the EORTC QLQ-C30 (*n* = 5). The most translated languages were isiXhosa (*n* = 21), Afrikaans (*n* = 21) and isiZulu (*n* = 17).

When translated by the investigators, no instrument met all the translation methodology criteria, but this analysis was often hampered by a lack of detailed reporting. In contrast, almost all the instrument developer versions met the complete set of recommended translation criteria used in this study. It was observed that translations produced by the European Organisation for Research and Treatment of Cancer or on their behalf, do not require professional translators for the forward translation step^20,21^ and the PedsQL^TM^ only required one backward translation.^22^ These instruments were therefore judged to not meet our study’s translation criteria. Only the instrument developers’ versions of the EQ-5D-3L (Afrikaans, isiZulu, isiXhosa, Sesotho and Setswana) and -5L (isiXhosa), SF-36 version 2 (Afrikaans, isiZulu, Sesotho) and SF-12 (isiXhosa) fully met the translation criteria.

As reported in Table 3, the stages reported most often in accordance with the translation criteria (i.e. a rating of ‘+’) were the pilot and cognitive debriefing stage (*n* = 32), review and harmonisation stage (*n* = 28), and the reconciliation and consensus stage (*n* = 25). Forward and back translations were often not performed in accordance with the recommendations (i.e. a rating of ‘–’) (both *n* = 8). Lack of any reporting details (i.e. a rating of ‘0’) occurred most often for the reconciliation and consensus stage after forward translation and the finalisation of the instrument through proofreading (both *n* = 11).

**TABLE 3:** Stages in instrument translation methodology.

Instrument and version	Instrument language(s)	Formal translation available from developer or investigator’s translation used?	Two parallel forward translations	Reconciliation and consensus	Two back translations	Review and harmonisation	Piloting and cognitive debriefing	Finalisation
**Generic instruments**								
AQOL-6D	Afrikaans, Setswana	No translation available from developer, translated by study investigator^23^	0	0	–	0	0	0
EQ-5D								
3L	Afrikaans	Developer’s translation used^24,25,26,27^	+	+	+	+	+	+
		Translated by investigator despite availability of official translation^23^	0	0	?	0	0	0
	isiXhosa	Translated by investigator using developer’s protocol^13^	+	+	+	+	+	+
		Developer’s translation used^14,24,25,26,27,28,29,30,31,32,33,34^	+	+	+	+	+	+
	isiZulu	Developer’s translation used^25,26,32^	+	+	+	+	+	+
	Sesotho	Developer’s translation used^26,32,35,36^	+	+	+	+	+	+
	Setswana	Developer’s translation used^26,32^	+	+	+	+	+	+
		Translated by investigator, unclear if the official translated version was available at time of study^23^	0	0	–	0	0	0
5L	isiXhosa	Developer’s translation used^33^	+	+	+	+	+	+
HUI3	Afrikaans	Translated by investigator, unclear if the official translated version was available at time of study^23^	0	0	–	0	0	0
	Setswana	No translation available from developer, translated by study investigator^23^	0	0	–	0	0	0
PedsQL 4.0 Generic Core Scale	Afrikaans, Sesotho, isiXhosa, isiZulu	Developer’s translation used^37^	+	+	–	+	+	+
Satisfaction with Life Scale	Afrikaans	No translation available from developer, translated by study investigator^38^	?	0	–	?	+	0
	Setswana	No translation available from developer at time of study, translated by study investigator^9^	?	?	?	?	+	?
**SF-36**								
Version 1	Afrikaans, isiXhosa	No translation available from developer, translated by study investigator^10^	0	0	0	0	+	0
	isiZulu	No translation available from developer, translated by study investigator^39^	–	0	–	0	0	0
		No translation available from developer, translated by study investigator^40^	–	0	?	0	?	0
Version 2	Afrikaans	Developer’s translation used^26,41,42,43^	+	+	+	+	+	+
	isiZulu	Developer’s translation used^26,44,45,46^	+	+	+	+	+	+
		Translated by investigator, unclear if the official translated version was available at time of study^47^	–	?	0	0	0	0
	Sesotho	Developer’s translation used^26,45^	+	+	+	+	+	+
SF-12	isiXhosa	Developer’s translation used^33^	+	+	+	+	+	+
WHOQOL-BREF	Afrikaans	No translated version available at the time of the study, translated by the study investigator^48^	–	0	–	+	0	0
		Translated by investigator, unclear if the official translated version was available at time of study^49^	–	0	0	0	+	0
	Setswana	No translated version available at the time of the study, translated by the study investigator^49^	–	0	0	0	+	0
**Disease-specific instruments**								
DLQI	Afrikaans, isiXhosa	No translated version available at the time of the study, translated by the study investigator^50^	?	0	?	+	+	0
EORTC QLQ-C30	Afrikaans, isiXhosa	Developer’s translation used^51,52,53^	?	+	?	+	+	?
	Sesotho	Developer’s translation used^53^
	Setswana	No translation available from developer, translated by study investigator^54^	–	0	0	0	0	0
	isiZulu	Translated by investigator despite availability of official translation^54^	–	0	0	0	0	0
		Translated by investigator despite availability of official translation^55^	?	0	0	0	0	0
		Developer’s translation used^53^	?	+	+	+	+	?
**FACT**								
General	Sepedi, Setswana, isiZulu	No translated version available at the time of the study, translated by the study investigator^11^	–	?	–	+	+	+
Breast	Sepedi, Setswana, isiZulu	No translated version available at the time of the study, translated by the study investigator^11^	–	?	–	+	+	+
PDQ-39	Afrikaans	Translated by investigator, unclear if the official translated version was available at time of study^12^	–	0	–	0	+	–
	isiZulu, Setswana	No translated version available at the time of the study, translated by the study investigator^12^	–	0	–	0	+	–

3L, 3-levels; 5L, 5-levels; AQoL-6D, Assessment of Quality of Life-6 domains; HUI3, Health Utility Index mark 3; PedsQL, Paediatric Quality of Life Inventory; SF, Short Form; WHOQOL-BREF, World Health Organization abbreviated version; DLQI, Dermatology Life Quality Index; EORTC QLQ-C30, European Organisation for Research and Treatment of Cancer Quality of Life Questionnaire Core Questionnaire; FACT, Functional Assessment of Cancer Therapy; PDQ-39, Parkinson’s Disease Questionnaire.

Rating: positive (+): procedure performed according to the quality criteria used; negative (–): procedure was not performed as recommended; uncertain (?): insufficient information available to rate the stage; unknown (0): no information available to rate the stage.

Common pitfalls by all investigators who conducted their own translations were producing only one forward or backward translation or using bilingual or native language speaking academics, research assistants or healthcare workers rather than employing professional translators. In addition, none reported the use of reconciliation and consensus stage and only one reported how the instrument was finalised. Half of investigators did however report the use of a pilot testing phase.

## Discussion

To the best of the authors’ knowledge, this is the first review and assessment of the translation quality of HRQoL instruments used in South Africa. It identified several HRQoL instruments suitable for CUAs that have been used in South Africa (Table 2). However, few have been translated for use in the South African population according to good practice guidelines (Table 3). Encouragingly, where reported, most studies used a language version in one of the three most spoken home languages, namely isiXhosa, Afrikaans and isiZulu, which together represents just over 50% of the population.^56^ Of the studies included in this review, only the instrument developers’ language versions of the EQ-5D-3L and -5L, SF-36 version 2 and SF-12 fully met the translation criteria used in the analysis. Of these, the EQ-5D-3L is currently the best placed of the existing HRQoL instruments suitable for CUAs for use in South Africa. This is based on the following factors: (1) It allows use and comparisons of results across multiple diseases (and is therefore preferred by many HTA agencies supporting healthcare priority setting and funding decisions^6^), (2) It was shown in this study to meet the standards for translation of PROMS, (3) It was found to have the most translations available of those studies included in the current analysis and (4) It has been used in a wide range of settings, populations and diseases in South Africa.^17^ The SF-36 and the EORTC QLQ-C30 were also frequently used but the EORTC QLQ-C30 did not meet the review’s translation criteria.

Within the context of using the studies for clinical and priority setting decisions in a multi-cultural country, it was concerning that only 39 out of the initial 144 studies included (27.1%) reported the use of translated instrument versions. However, this is higher than that reported by Bello et al.^57^ Only 14.0% of the studies in their systematic review on the properties of stroke quality of life outcomes measures in Africa used translated instruments. The remainder of the findings is consistent with the reported literature. A 2003 systematic review of the translation and adaptation of generic HRQoL measures in Africa, Asia, Eastern Europe, the Middle East and South America highlighted that in the 1990s several publications raised concern about the quality of translated versions of HRQoL instruments, focusing on the quality of the methodology.^58^ They found very few studies that reported that the instruments were assessed for equivalence during translation and none considered it in any detail. The authors concluded that more work needs to be performed to improve translations. Yet despite several decades of experience and numerous translation and adaptation frameworks, guidelines and good practice principles^59,60,61^ problems remain with instrument translation into non-English languages. For example, more recently Al Sayah et al.^62^ also observed that reporting details were lacking in Arabic translations and cross-cultural adaptations of HRQoL instruments. Consequently, the authors could not evaluate the translation quality of most of the instruments identified. Reporting details were also lacking for Brazilian Portuguese generic and cancer specific PROMs, and most problems were with the forward translation, back translation and expert review steps.^63^ This study’s finding that there was a lack of a pilot and cognitive debriefing step by half of investigators who created their own translations is also not unique: a systematic review of childhood HRQoL measures in sub-Saharan Africa found only two instruments (out of 10 identified) that attempted to establish cross-cultural adaptation through linguistic and conceptual equivalence testing.^64^ Although not explored in these analyses, it is worth reflecting on the possible reasons for the lack of improvement in translation quality. For instance, the importance of high-quality translations and impact of poor translations and adaptations of instruments outside of these countries may not receive the necessary attention in the literature read by researchers and clinicians interested in HRQoL. Certainly, HRQoL research between 2000 and 2019 were predominantly conducted in North America and Europe, and the studies were published in English language journals^65^ (which are owned by publishing companies based in these regions). Furthermore, researchers in low- and middle-income countries may also not have the resources available to conduct translations according to the guidelines and the guidelines themselves may not account for the complexities. These last two points were identified and discussed in detail by De Wet et al. in a case study of their study’s research documents that were translated into isiXhosa,^66^ and were also highlighted in some of the publications included in this review.^11,13^

Finally, the studies included in this review were mainly in one disease area (HIV). The 2019 review also found a strong focus on HIV and showed that HRQoL data in chronic conditions that contributed the most to the country’s burden of disease, were lacking.^17^ Given the existing requirement in the SAGPS and the draft NEML HTA guidelines to use a South African validated HRQoL instrument for economic evaluations and the gaps identified in this and 2019 review, further work may be needed if CUA has to form the basis of HTA under NHI. For example, if the EQ-5D were to be used for HTA under NHI, a South African value set will be needed. Until such a value set is created, one from another country must be used, which increases uncertainty in CUA results and limits health policy decisions.

Measuring patient outcomes is the basis for decisions about the most appropriate treatment for a patient, tracking healthcare quality, evaluating service performance, and priority setting such as funding of cost-effective treatments. Consequently, this work has several implications for clinical practice, health policy and HRQoL research. Without using HRQoL measures in the target patient and population’s native language or instruments translated using sound methodologies, it cannot be certain that the instruments accurately evaluate the patients’ views about their health and subsequent health outcomes. This brings into question the validity of the results obtained and compromises the instrument’s use in clinical practice and for health policy decisions. Using poorly translated instruments also contribute to wasteful and inefficient resources use when time and money are spent on administering an instrument that may produce unreliable results. This review therefore makes several recommendations based on the study’s findings and existing literature (Table 4) specific to HRQoL measurement in any multi-cultural setting.

**TABLE 4:** Recommendations for conducting health-related quality of life research and using health-related quality of life instruments for clinical and policy decision-making in multicultural settings.

Who	What	Explanation
Researchers and research organisations/clinicians and clinical organisations	Use HRQoL measurements with existing translated versions available from instrument developers regardless of the purpose.	Evidence from this study suggests that such measurements have likely been created according to best practices that reflect standardised and tested approaches, thus ensuring the instrument is valid in the target population.
Researchers and research organisations/clinicians and clinical organisations	In the absence of a translated version from the developer, retrieve and employ the translation methods suggested by the developer and, ideally, getting their support or input into the process.	This could maintain the validity of the adapted measure because the evidence from this review suggests that developer’s methods are most likely to conform to current best practice. Instrument developers are also likely to have access to individuals or organisations who can contribute towards the process.
Researchers and research organisations/clinicians and clinical organisations	When translating an existing instrument, consult and follow guidelines and best practice documents that report recommended PROM translation methods.	These documents reflect consensus views on acceptable, standardised methods^19^ and are likely to meet regulatory and HTA agencies’ requirements for validated PROMs relevant to the local context.
Researchers and research organisations/clinicians and clinical organisations	For generating HRQoL data for CUAs choose generic HRQoL MAUIs.	Health technology assessment agencies and funding decision-makers prefer generic MAUI over disease specific instruments.^6^ In addition, disease specific or non-MAUIs will require mapping to MAUIs, which increases uncertainty in the CUA results.^7^
Instrument developers	Provide user-support guides such as translation manuals and/or provide contact information to alert researchers intending to translate instruments of the interest and availability to work collaboratively.	This would support the creation of valid instruments and as suggested by this review, is likely to increase the use of the instrument in populations speaking the target language compared with instruments that require translation by the study investigator.
Health policy and funding decision makers	Require that the HRQoL instruments be valid in the local context and provide guidance on how to establish validity.	Local data will be needed for economic evaluations,^16^ but guidance on what constitutes a validated instrument for such purposes is currently lacking from the NHI and National Department of Health. Such guidance will support the generation of HRQoL data for HTA and strengthen the level of confidence that decisions are evidence based and relevant to the local context.
Health policy and funding decision makers	Provide clear guidance on which HRQoL instruments will be needed for decision-making and how to create such instruments if they do not currently exist in a suitable form.	Evidence from this review showed that there are a range of HRQoL instruments suitable for CUAs in South Africa, but not all may be valid in the local context because of the translation methods used. Whilst most translation methods for PROM recommended by guidelines would achieve comparable results,^59^ South African specific guidance on choice of HRQoL instrument for HTA and how to create such instrument would provide clarity to researchers, avoid wasteful research and prevent any decision that is not evidence based or relevant to the local context.

HRQoL, health-related quality of life; PROM, Patient reported outcomes measures; HTA, health technology assessment; CUA, cost-utility analyse; MAUI, multi-attribute utility instruments; NHI, National Health Insurance.

For South Africa, where national HTA and economic evaluations are going to play a more important role in sustainable patient access to health technologies, both patients and funders would benefit from incorporating the recommendations presented in Table 4. This is because providing high quality HRQoL data for new technologies could get patients faster access to new technologies, whilst both private and public funders will have more certainty that the introduction of technologies is proven value for money.

## Strengths and limitations

The analysis was based on a comprehensive literature review, the foundation of which was a 2019 systematic review. However, only one researcher was involved in extracting the translation methods from the publications, which may have resulted in errors. To mitigate this, the extraction form used in Excel^®^ served as a checklist, and the articles were re-evaluated on more than three separate occasions. It is acknowledged that various translation frameworks exist and that the choice of Guillemin et al. and Wild et al. may be considered arbitrary. However, evidence is lacking on the best methods for translation and cross-cultural adaptation and most translation methods recommended by guidelines would achieve comparable results.^59^ It is therefore unlikely that the results of this review would have been much different if other translation criteria were used. In addition, the use of publications to measure the quality of research is limited by the information provided in the article and it is therefore possible that the methodology in the studies did follow recommended translation methods but was simply poorly reported. Lastly, the analysis focussed on HRQoL instruments suitable for CUAs, thus it is not a comprehensive review of all PROMs used in South Africa.

## Conclusion

The EQ-5D-3L may be best suited for use in South Africa where data are needed for a CUA. However, further work and detailed guidance from the National Department of Health on the most suitable HRQoL instrument for the South African context will be needed if the CUAs will be required as part of the HTA process under NHI. Acting upon the recommendations from this study could result in more robust measurement of HRQoL in South Africa and more informed decisions on the introduction and use of health technologies from both a patient and national healthcare policy standpoint. The recommendations on translation methodology quality are also relevant to clinicians wanting to obtain reliable health outcomes data from the patients’ perspective.

## References

[1] National Department of Health. National health insurance bill. Pretoria: Government Gazette, 2019;(42598), p. 60.

[2] Solanki GC, Cornell JE, Besada D, Morar RL, Wilkinson T. The competition commission health market inquiry report: An overview and key imperatives. S Afr Med J. 2020;110(2):88–91. 10.7196/SAMJ.2020.v110i2.14455 32657675

[3] Mash R. National health insurance unpacked: Part 4: Remuneration of practitioners. S Afr Fam Pract. 2020;62(1):e1–e2. 10.4102/safp.v62i1.5241 PMC8378206 33179958

[4] Competition Commission South Africa. Health market inquiry: Final findings and recommendations report. Pretoria: Competition Commission South Africa; 2019.

[5] Essential Drugs Programme. Health technology assessment methods guide to inform the selection of medicines to the South African national essential medicines list. 14 June 2021. Pretoria: National Department of Health, 2021; p. 68.

[6] Brazier J, Ara R, Rowen D, Chevrou-Severac H. A review of generic preference-based measures for use in cost-effectiveness models. Pharmacoeconomics. 2017;35(1):21–31. 10.1007/s40273-017-0545-x 29052157

[7] Mukuria C, Rowen D, Harnan S, et al. An updated systematic review of studies mapping (or cross-walking) measures of health-related quality of life to generic preference-based measures to generate utility values. Appl Health Econ Health Policy. 2019;17(3):295–313. 10.1007/s40258-019-00467-6 30945127

[8] Guillemin F, Bombardier C, Beaton D. Cross-cultural adaptation of health-related quality of life measures: Literature review and proposed guidelines. J Clin Epidemiol. 1993;46(12):1417–1432. 10.1016/0895-4356(93)90142-N 8263569

[9] Wissing MP, Thekiso SM, Stapelberg R, et al. Validation of three Setswana measures for psychological wellbeing. SA J Ind Psychol. 2010;36(2):1–8. 10.4102/sajip.v36i2.860

[10] Okeefe EA, Wood R. The impact of HIV infection on quality of life in a multiracial South African population. Qual Life Res. 1996;5(2):275–280. 10.1007/BF00434749 8998496

[11] Mullin V, Cella D, Chang CH, et al. Development of three African language translations of the FACT-G. Qual Life Res. 2000;9(2):139–149. 10.1023/A:1008903304950 10983478

[12] Nelson G, Ndlovu N, Christofides N, Hlungwani TM, Faust I, Racette BA. Validation of Parkinson’s disease-related questionnaires in South Africa. Parkinsons Dis. 2020;2020:9. 10.1155/2020/7542138 PMC7306845 32617145

[13] Mkoka S, Vaughan J, Wylie T, Yelland H, Jelsma J. The pitfalls of translation – A case study based on the translation of the EQ5D into Xhosa. S Afr Med J. 2003;93(4):265–266. 12806712

[14] Jelsma J, Mkoka S, Amosun SL, Nieuwveldt J. The reliability and validity of the Xhosa version of the EQ5D. Disabil Rehabil. 2004;26(2):103–108. 10.1080/09638280310001629705 14668147

[15] Akehurst RL, Abadie E, Renaudin N, Sarkozy F. Variation in health technology assessment and reimbursement processes in Europe. Value Health. 2017;20(1):67–76. 10.1016/j.jval.2016.08.725 28212972

[16] National Department of Health. Medicines and Related Substances Act (10 of 1965) regulations relating to a transparent pricing system for medicines and scheduled substances: Publication of the guidelines for pharmacoeconomic submissions. Pretoria: Government Gazette, 2013; p. 69.

[17] Marsh SE, Truter I. Fit for the future? Status of health-related quality of life research in South Africa. Int J Technol Assess Health Care. 2020;36(5):508–517. 32988423 10.1017/S0266462320000690

[18] Marsh SE. Making connections and measuring performance: Bibliometric analysis of multi-attribute, preference-based health-related quality of life research in South Africa. Value Health Reg Issues. 2020;22:99–107. 10.1016/j.vhri.2020.07.574 32823062

[19] Wild D, Grove A, Martin M, et al. Principles of good practice for the translation and cultural adaptation process for Patient-Reported Outcomes (PRO) measures: Report of the ISPOR task force for translation and cultural adaptation. Value Health. 2005;8(2):94–104. 10.1111/j.1524-4733.2005.04054.x 15804318

[20] Cull A, Sprangers M, Bjordal K, Aaronson N, West K, Bottomley A. EORTC quality of life group translation procedure. Brussels: EORTC; 2002.

[21] Kuliś D, Bottomley A, Velikova G, Greimel E, Koller M. EORTC quality of life group translation procedure. Brussels: EORTC Quality of Life Group; 2017.

[22] Linguistic validation of the PedsQL™ – A quality of life questionnaire [homepage on the Internet]. 2002 [cited 2020 Nov 11]. Available from: https://www.pedsql.org/translations.html

[23] Gow J, George G, Govender K. A comparison of QOL between HIV positive and negative diamond miners in South Africa. Sahara J-J Soc Asp HIV/AIDS. 2013;10(2):89–95. 10.1080/17290376.2013.870066 PMC3914423 24405284

[24] Jelsma J, Ferguson GD. The determinants of self-reported HRQOL in a culturally and socially diverse South African community. Bull World Health Organ. 2004;82(3):206–212. 15112009 PMC2585936

[25] Wouters E, Meulemans H, Van Rensburg HCJ, Heunis C, Mortelmans D. Short-term physical and emotional health outcomes of public sector antiretroviral therapy in the Free State province of South Africa. Qual Life Res. 2007;16(9):1461–1471. 10.1007/s11136-007-9260-y 17899446

[26] Van Aartsen J, Van Aswegen H. Changes in biopsychosocial outcomes for a mixed cohort of intensive care unit survivors. South Afr J Physiother. 2018;74(1):10. 10.4102/sajp.v74i1.427 PMC6093101 30135920

[27] Elwell-Sutton T, Folb N, Clark A, Fairall L, Lund C, Bachmann MO. Socioeconomic position and depression in South African adults with long-term health conditions: A longitudinal study of causal pathways. Epidemiol Psychiatr Sci. 2019;28(2):199. 10.1017/S2045796017000427 28805174 PMC6998924

[28] Hughes J, Jelsma J, Maclean E, Darder M, Tinise X. The HRQOL of people living with HIV/AIDS. Disabil Rehabil. 2004;26(6):371–376. 10.1080/09638280410001662932 15204489

[29] Jelsma J, Maclean E, Hughes J, Tinise X, Darder M. An investigation into the HRQOL of individuals living with HIV who are receiving HAART. AIDS Care-Psychol Socio-Med Asp AIDS-HIV. 2005;17(5):579–588. 10.1080/09540120412331319714 16036244

[30] Jelsma J, Maart S, Eide A, Ka’Toni M, Loeb M. The determinants of HRQOL in urban and rural isiXhosa-speaking people with disabilities. Int J Rehabil Res. 2007;30(2):119–126. 10.1097/MRR.0b013e32813a2e88 17473623

[31] Parker R, Jelsma J, Stein DJ. Pain in amaXhosa women living with HIV/AIDS: Translation and validation of the BPI-Xhosa. J Pain Symptom Manage. 2016;51(1):126–132. 10.1016/j.jpainsymman.2015.08.004 26344550

[32] Pillay P, Wadley AL, Cherry CL, Karstaedt AS, Kamerman PR. Psychological factors associated with painful versus non-painful HIV-associated sensory neuropathy. AIDS Behav. 2018;22(5):1584–1595. 10.1007/s10461-017-1856-9 28710709

[33] Burnand HG, McMahon SE, Sayers A, et al. The EQ-5D-3L administered by text message compared to the paper version for hard-to-reach populations in a rural South African trauma setting: A measurement equivalence study. Arch Orthop Trauma Surg. 2020;141:947–957. 10.1007/s00402-020-03574-5 32785761 PMC8139899

[34] Jackson K, Wadley AL, Parker R. Managing pain in HIV/AIDS: A therapeutic relationship is as effective as an exercise and education intervention for rural amaXhosa women in South Africa. BMC Public Health. 2021;21(1):1–14. 10.1186/s12889-021-10309-7 33546647 PMC7866667

[35] Barnes R, Jelsma J, Parker R. Joint pain within adult middle-aged women, attending a community clinic in a peri-urban area in South Africa: A cross-sectional survey. Disabil Rehabil. 2018;41(11):1343–1350. 10.1080/09638288.2018.1428368 29347849

[36] Parker R, Barnes RY, Jelsma J. Improvements in health-related quality of life and function in middle-aged women with chronic diseases of lifestyle after participating in a non-pharmacological intervention programme: A pragmatic randomised controlled trial. Afr J Disab. 2019;8(1):1–14. 10.4102/ajod.v8i0.428 PMC6424002 30899683

[37] Shiau S, Evans H, Strehlau R, et al. Behavioral functioning and quality of life in South African children living with HIV on antiretroviral therapy. J Pediatr. 2020;227:308–313. e2. 10.1016/j.jpeds.2020.07.057 32712285 PMC8811608

[38] Wissing MP, Van Eeden C. Empirical clarification of the nature of psychological well-being. S Afr J Psychol. 2002;32(1):32–44. 10.1177/008124630203200105

[39] McInerney PA, Nicholas PK, Wantland D, et al. Characteristics of anti-tuberculosis medication adherence in South Africa. Appl Nurs Res. 2007;20(4):164–170. 10.1016/j.apnr.2006.06.006 17996802

[40] McInerney PA, Ncama BP, Wantland D, et al. QOL and physical functioning in HIV-infected individuals receiving antiretroviral therapy in KwaZulu-Natal, South Africa. Nurs Health Sci. 2008;10(4):266–272. 10.1111/j.1442-2018.2008.00410.x 19128302

[41] Rensburg CJV, Kulich KR, Carlsson J, Wiklund IK. What is the burden of illness in patients with reflux disease in South Africa? S Afr Gastroenterol Rev. 2005;3(3):16–21.

[42] Kulich KR, Madisch A, Pacini F, et al. Reliability and validity of the GSRS and QOLRAD questionnaire in dyspepsia: A six-country study. Health Qual Life Outcomes. 2008;6:12. 10.1186/1477-7525-6-12 18237386 PMC2276197

[43] Karachi F, Hanekom S, Faure M. HRQOL of patients 12 months following surgical intensive care discharge. S Afr J Physiother. 2011;67(1):28–34. 10.4102/sajp.v67i1.36

[44] Kuo C, Operario D. Health of adults caring for orphaned children in an HIV-endemic community in South Africa. AIDS Care-Psychol Socio-Med Asp AIDS-HIV. 2011;23(9):1128–1135. 10.1080/09540121.2011.554527 PMC3139727 21480009

[45] Odek WO. Formal employment and HRQOL among people living with HIV in South Africa. Appl Res Qual Life. 2013;8(2):145–168. 10.1007/s11482-012-9183-9

[46] Reddy P, Frantz JM. The QOL of HIV-infected and non-infected women post-caesarean section delivery. Health SA Gesondheid. 2017;22:87–92. 10.1016/j.hsag.2016.09.006

[47] Nair KM, Muthukrishna N. Psychological well-being and HRQOL among a group of low-income women living with HIV/AIDS in South Africa. J Psychol Afr. 2009;19(4):517–529. 10.1080/14330237.2009.10820324

[48] Badenhorst M, Brown JC, Lambert MI, Van Mechelen W, Verhagen E. QOL among individuals with rugby-related spinal cord injuries in South Africa: A descriptive cross-sectional study. BMJ Open. 2018;8(6):e020890. 10.1136/bmjopen-2017-020890 PMC6045750 29961017

[49] Govender SM, De Jongh M. Identifying hearing impairment and the associated impact on the quality of life among the elderly residing in retirement homes in Pretoria, South Africa. S Afr J of Commun Disord. 2021;68(1):1–9. 10.4102/sajcd.v68i1.788 PMC8008190 33764149

[50] Jobanputra R, Bachmann MO. The effect of skin diseases on QOL in patients from different social and ethnic groups in Cape Town, South Africa. Int J Dermatol. 2000;39(11):826–831. 10.1046/j.1365-4362.2000.00073.x 11123442

[51] Du Toit G, Kidd M. Contextual quality of life of HIV-positive patients with cervical carcinoma at Tygerberg Hospital. S Afr J Gynaecol Oncol. 2013;5(2):41–46. 10.1080/20742835.2013.11441208

[52] Du Toit GC, Kidd M. An analysis of the psychometric properties of the translated versions of the EORTC QLQ-CX24 in the two South African indigenous languages of Xhosa and Afrikaans. Eur J Cancer Care. 2016;25(5):832–838. 10.1111/ecc.12333 26052872

[53] Minnaar CA, Kotzen JA, Naidoo T, et al. Analysis of the effects of mEHT on the treatment-related toxicity and quality of life of HIV-positive cervical cancer patients. Int J Hyperth. 2020;37(1):263–272. 10.1080/02656736.2020.1737253 32180481

[54] Jeppe CY, Becker P, Smith MD. Post-Frey procedure QOL in South African patients with painful chronic pancreatitis. JOP. 2013;14(1):21–30. 23306331 10.6092/1590-8577/933

[55] Shaik F, Uldrick TS, Esterhuizen T, Mosam A. HRQoL in patients treated with antiretroviral therapy only versus chemotherapy and antiretroviral therapy for HIV-associated Kaposi Sarcoma: A randomised control trial. J Glob Oncol. 2018;4:9. 10.1200/JGO.18.00105 PMC6818281 30354935

[56] Government Communications. Official guide to South Africa 2018/19. 16th ed. Pretoria: Government Communications, 2019; p. 292.

[57] Bello UM, Chutiyami M, Salihu D, et al. Quality of life of stroke survivors in Africa: A systematic review and meta-analysis. Qual Life Res. 2021;30(1):1–19. 10.1007/s11136-020-02591-6 32712933

[58] Bowden A, Fox-Rushby JA. A systematic and critical review of the process of translation and adaptation of generic health-related quality of life measures in Africa, Asia, Eastern Europe, the Middle East, South America. Soc Sci Med. 2003;57(7):1289–1306. 10.1016/S0277-9536(02)00503-8 12899911

[59] Epstein J, Santo RM, Guillemin F. A review of guidelines for cross-cultural adaptation of questionnaires could not bring out a consensus. J Clin Epidemiol. 2015;68(4):435–441. 10.1016/j.jclinepi.2014.11.021 25698408

[60] Eremenco S, Pease S, Mann S, Berry P, Subcommittee PROCsP. Patient-Reported Outcome (PRO) consortium translation process: Consensus development of updated best practices. J Patient Rep Outcomes. 2017;2(1):12. 10.1186/s41687-018-0037-6 29757299 PMC5934912

[61] Valdez D, Montenegro MS, Crawford BL, Turner RC, Lo W-J, Jozkowski KN. Translation frameworks and questionnaire design approaches as a component of health research and practice: A discussion and taxonomy of popular translation frameworks and questionnaire design approaches. Soc Sci Med. 2021;278:113931. 10.1016/j.socscimed.2021.113931 33905986

[62] Al Sayah F, Ishaque S, Lau D, Johnson JA. Health related quality of life measures in Arabic speaking populations: A systematic review on cross-cultural adaptation and measurement properties. Qual Life Res. 2013;22(1):213–229. 10.1007/s11136-012-0129-3 22350531

[63] Albach CA, Wagland R, Hunt KJ. Cross-cultural adaptation and measurement properties of generic and cancer-related patient-reported outcome measures (PROMs) for use with cancer patients in Brazil: A systematic review. Qual Life Res. 2018;27(4):857–870. 10.1007/s11136-017-1703-5 28887596 PMC5874274

[64] Ngwira LG, Khan K, Maheswaran H, et al. A systematic literature review of preference-based health-related quality-of-life measures applied and validated for use in childhood and adolescent populations in sub-Saharan Africa. Value Health Reg Issues. 2021;25:37–47. 10.1016/j.vhri.2020.11.009 33765659

[65] Zheng S, He A, Yu Y, Jiang L, Liang J, Wang P. Research trends and hotspots of health-related quality of life: A bibliometric analysis from 2000 to 2019. Health Qual Life Outcomes. 2021;19(1):1–13. 10.1186/s12955-021-01767-z 33892744 PMC8063463

[66] De Wet A, Dowling T, Swartz L, et al. Complexities in the process of translating research documents in cross-cultural settings. Global Public Health. 2020;15(6):818–827. 10.1080/17441692.2020.1718736 31994442

